# Heat shock proteins in protein folding and reactivation

**DOI:** 10.18699/vjgb-25-02

**Published:** 2025-02

**Authors:** D. Malkeyeva, E.V. Kiseleva, S.A. Fedorova

**Affiliations:** Institute of Cytology and Genetics of the Siberian Branch of the Russian Academy of Sciences, Novosibirsk, Russia; Institute of Cytology and Genetics of the Siberian Branch of the Russian Academy of Sciences, Novosibirsk, Russia; Institute of Cytology and Genetics of the Siberian Branch of the Russian Academy of Sciences, Novosibirsk, Russia Kurchatov Genomic Center of ICG SB RAS, Novosibirsk, Russia

**Keywords:** heat shock proteins, molecular chaperones, protein folding, protein quality control, HSP, белки теплового шока, молекулярные шапероны, фолдинг белков, контроль качества белков, HSP

## Abstract

Throughout their lives, cells synthesise new and dispose of the old, denatured proteins and insoluble protein aggregates. An important role in maintaining proteostasis is played by chaperones, which fold various proteins and promote degradation of denatured or misfolded proteins via proteasomes or autophagy. Despite protein folding being an accurate process, as organisms age and experience stress, errors accumulate, which leads to the formation of protein aggregates that can result in pathological changes. In addition, stress factors such as elevated temperature and altered pH can promote protein denaturation that can result in the proteins not only losing their native functions, but also gaining novel cytotoxic properties. With the increase of human average lifespan, more and more cases of proteinopathies – diseases caused by disruptions in proteostasis, e. g. Alzheimer’s disease, Huntington’s disease etc. – emerge. Therefore, identification of mechanisms preventing the formation of cytotoxic protein aggregates and promoting their clearance is of high importance. Heat shock proteins (HSPs) are the molecular chaperones involved in folding nascent proteins and refolding the denatured ones, leading to their reactivation. Heat shock proteins vary in structure and functions and are found in all prokaryotes and eukaryotes discovered to date. HSPs are constantly synthesised in cells under normal conditions, and a multitude of them are dramatically up-regulated during stress, which includes heat shock (which earned them their name) and metabolic stress caused by the increased numbers of misfolded proteins. In this review, we describe mechanisms of action and functions of members of five heat shock protein families.

## Abstract (ru)

В процессе жизнедеятельности в каждой клетке происходят синтез новых белков и удаление старых, денатурированных белков и нерастворимых белковых агрегатов. В поддержании протеостаза значительную роль играют шапероны, которые участвуют в придании правильной конформации (фолдинге) многих белков и способствуют деградации денатурированных или неправильно свернутых белков посредством протеаз или аутофагии. Несмотря на то что фолдинг белков – довольно точный процесс, с возрастом и под воздействием стресса накапливаются ошибки, приводящие к образованию нерастворимых белковых агрегатов, которые могут вызывать различные патологии. Воздействие стрессовых факторов, таких как повышенная температура и изменение кислотности среды, также может способствовать изменению нативной конформации белков, в результате чего они могут не только терять выполняемые в норме функции, но и приобретать новые цитотоксические свойства. В связи с увеличением средней продолжительности жизни человека в мире отмечается рост протеинопатий – заболеваний, связанных с нарушением протеостаза, к которым относятся, например, болезни Альцгеймера, Паркинсона, Хантингтона; поэтому выявление механизмов, препятствующих накоплению и способствующих удалению цитотоксичных агрегатов, стало актуальной задачей. Белки теплового шока (heat shock proteins, HSP) – молекулярные шапероны, принимающие участие как в придании правильной конформации вновь синтезированным белкам, так и в рефолдинге денатурированных белков с их последующей реактивацией. HSP разнообразны по структуре и выполняемым функциям и встречаются у всех изученных про- и эукариотических организмов. HSP синтезируются в клетке постоянно. Выработка множества из них многократно усиливается при стрессах, включая тепловой (за что они и получили свое название) и метаболический стресс, возникающий из-за повышения количества неправильно свернутых белков. В настоящем обзоре описаны механизмы действия и функции представителей пяти семейств HSP в фолдинге и реактивации белков

## Introduction

Heat shock proteins (molecular chaperones, HSPs) are a class of conserved proteins, the main function of which is protein quality control (van Leeuwen, Kampinga, 2018). Heat shock proteins are present in all prokaryotes and eukaryotes discovered to date (Lindquist, 1986). The majority of HSPs are synthesised under normal conditions and are up-regulated during stress, e. g. hyper- or hypothermia, hypoxia, oxidative stress, and infection (Sørensen et al., 2003; Kampinga et al., 2009; Sarkar et al., 2011). Under normal conditions, HSPs serve as “molecular chaperones” by folding nascent proteins (Ellis, 1987; Feder, Hofmann, 1999). During stress, the redox balance and hydration of the cell can be disrupted, which leads to protein misfolding (Jolly, Morimoto, 2000). Misfolded proteins, in turn, can gain deleterious functions and tend to form insoluble aggregates (Jolly, Morimoto, 2000). The induction of HSP synthesis allows the cells to maintain homeostasis due to their ability to refold the damaged proteins, prevent aggregation, and to resolubilise the already formed protein aggregates (Jolly, Morimoto, 2000). Mutations in certain HSPs result in disorders, such as myopathies, neuropathies, and lens or retinal diseases (Macario et al., 2005; Kakkar et al., 2014).

Based on their structure and functions, HSPs are classified into five major families, with their names reflecting the members’ molecular mass in kilodaltons (kDa): Hsp100 (or Hsp110), Hsp90, Hsp70, Hsp60, and small HSPs (sHSPs, up to 43 kDa) (Sarkar et al., 2011; Bar-Lavan et al., 2016). HSPs cooperate with co-chaperones Hsp40, Hsp10, and NEF, which are sometimes placed into separate HSP families. For human HSP families, a standard nomenclature was suggested in 2009 by Professor H.H. Kampinga et al.: HSPH (Hsp110), HSPC (Hsp90), HSPA (Hsp70), DNAJ (Hsp40), HSPB (sHSPs), and chaperonins HSPD/E (HSP60/HSP10) and CCT (TRiC) (Kampinga et al., 2009).

## Hsp100

This family of HSPs (Clp in bacteria, HSPH in humans) includes 100–110 kDa chaperones capable of proteolysis and protein aggregate degradation (Sarkar et al., 2011; Mogk et al., 2015). These chaperones are abundant in prokaryotes and are present in unicellular eukaryotes (e. g. Hsp104 and Hsp78 in budding yeast, Saccharomyces cerevisiae); in multicellular organisms, Hsp100 can only be found in mitochondria (Sarkar et al., 2011). Hsp100 proteins belong to the AAA+ (ATPases associated with diverse cellular activities) superfamily and share the AAA domain defined by a region of ~230 amino acid residues containing Walker A, Walker B, sensor-1, and sensor-2 motifs that are necessary for nucleotide binding and hydrolysis and for Hsp100 oligomerisation resulting in ringlike structures (Mogk et al., 2015; Mokry et al., 2015). Depending on the number of the AAA domains, Hsp100 proteins can be divided into two classes: Class I with two nucleotide binding domains, and Class II with one nucleotide binding domain (Hodson et al., 2012; Mokry et al., 2015). Class I includes ClpA, ClpB (Hsp104), and ClpC; Class II includes ClpX and HslU (Hodson et al., 2012).

All of the Hsp100 family members have an N-terminal domain contributing to the binding to protein aggregates (Mokry et al., 2015). Certain Hsp100 proteins have an M domain located within the first nucleotide binding domain. The flexible M domains are located on the outer surface of the Hsp100 ring and play an essential role in substrate interaction and protein aggregate reactivation (Mokry et al., 2015). In the presence of ATP, Hsp100 form ring-like homohexamers with a central pore of ~15 Å through which unfolded substrates trapped in protein aggregates are pulled (Hodson et al., 2012; Duran et al., 2017). Inside, the pore is lined with loops containing tyrosine residues that bind the aggregated peptides and translocate them through the channel by performing a rowing motion fuelled by ATP hydrolysis (Saibil, 2013). The substrate is pulled into the Hsp100 central pore as a loop, and not by its terminus, because internal segments of aggregated proteins are preferentially targeted (Avellaneda et al., 2020). Nevertheless, the insertion of the substrate into the channel with its free termini is also possible (Avellaneda et al., 2020).

Some of the Hsp100 family members are associated with proteases, and translocate the unfolded aggregate components to them for degradation (Hodson et al., 2012). For example, the bacterial ClpA, ClpC, and ClpX are associated with ClpP protease, and the HslU (ClpY) chaperone is associated with the HslV (ClpQ) protease (Hodson et al., 2012). The bacterial ClpB, the yeast Hsp78 and Hsp104, and the plant Hsp101 are not connected to any proteases and thus reactivate the aggregated proteins (Hodson et al., 2012).

Hsp100 can perform disaggregation independently; however, their activity is greatly enhanced by the presence of the Hsp70/Hsp40/NEF chaperone system (Mokry et al., 2015). For example, Hsp104 and ClpB are almost incapable of recognising aggregated proteins in the absence of Hsp70 (Mogk et al., 2015). Hsp70 chaperones bind to aggregated peptides and then directly interact with the Hsp100 M domains thereby presenting the substrate to the disaggregase. Next, the substrate is translocated through the Hsp100 central pore and unfolded (Mogk et al., 2015).

## Hsp90

Hsp90 proteins (HtpG in bacteria, HSPC in humans) are some of the most abundant chaperones, making up to 1–2 % of total proteins in eukaryotic cells (Sarkar et al., 2011; Li, Buchner, 2013). In addition to folding denatured proteins, Hsp90 facilitates maturation of various de novo synthesised peptides (Bar-Lavan et al., 2016). Bacteria have an Hsp90 protein (HtpG in Escherichia coli); no Hsp90 genes were found in Archaea (Li, Buchner, 2013). In yeast, two members of Hsp90 are present, namely, Hsc82 and Hsp82, that are localised to cytosol (Li, Buchner, 2013). Plants possess ch-Hsp90 found in chloroplasts (Li, Buchner, 2013). In the fruit fly Drosophila melanogaster, one Hsp90 member, i. e. Hsp83, has been discovered; it is the only HSP-coding gene in D. melanogaster that has an intron (Sarkar et al., 2011). In mammals, there are four members of the family, of which two isoforms of Hsp90 (Hsp90α and Hsp90β) are localised to the cytosol, Grp94 is located in the endoplasmic reticulum, and Trap-1 is present in the mitochondria (Sarkar et al., 2011; Li, Buchner, 2013).

Hsp90 proteins have three domains: the highly conserved N-terminal domain with the ATP binding site and a charged loop segment, an M domain necessary for substrate binding and regulation of ATP hydrolysis, and a C-terminal domain allowing for the dimerisation of Hsp90 proteins and interaction with several co-chaperones (Bar-Lavan et al., 2016). When it does not bind ATP, the Hsp90 homodimer adopts a V-shaped form, termed “open conformation” (Li, Buchner, 2013). ATP binding leads to a change in the N and M domains orientation and the transition of the homodimer to a “closed conformation”, with the N domains dimerised (Li, Buchner, 2013). After ATP hydrolysis, the N domains dissociate, and Hsp90 returns into the “open conformation” (Li, Buchner, 2013). The transition between the conformations is determined by Hsp90’s interaction with the client proteins and with its various co-chaperones (Li, Buchner, 2013; Bar-Lavan et al., 2016). Interaction with co-chaperones and substrates occurs when Hsp90 is in the “open conformation”; during the transition to the “closed conformation”, the substrate is folded; ADP, phosphate, the substrate, and co-chaperones are then released and Hsp90 returns into the “open conformation” (Li, Buchner, 2013).

Co-chaperone regulation is a conserved trait of the eukaryotic Hsp90 system. More than 20 Hsp90 co-chaperones are known to date (Li, Buchner, 2013). They regulate the Hsp90 function by inhibiting or activating the chaperone’s ATPase activity and recruiting client proteins. Different co-chaperones interact with each other to facilitate the Hsp90 client maturation, and the composition of the co-chaperone complexes depends on the client protein type (Li, Buchner, 2013).

Hsp90 are more specialised than other HSPs. Together with their co-chaperones, Hsp90 play an important role in folding of at least 200 different peptides under normal conditions and in refolding of denatured proteins following stress (Sarkar et al., 2011; Saibil, 2013). Among the Hsp90 clients are signalling proteins participating in cell cycle regulation, kinases, steroid hormone receptors, and the tumour suppressor p53 (Saibil, 2013).

## Hsp70

The members of the Hsp70 family (DnaK in prokaryotes, HSPA in humans) have the molecular mass of 70 kDa and are the most conserved of the HSPs. Thus, Hsp70 proteins of all characterized organisms share ~50 % amino acid identity (Sarkar et al., 2011; Bar-Lavan et al., 2016). A distinctive feature of this HSP family is the occurrence of multiple copies of Hsp70 genes in the majority of species (Sarkar et al., 2011). For example, the yeast S. cerevisiae has 14 Hsp70 copies (Sarkar et al., 2011); the fruit fly D. melanogaster has 6 almost identical stress-inducible Hsp70 genes, stress-inducible gene Hsp68, and several constantly expressed Hsc70 (Heat shock cognate 70) genes (Tower, 2011; Xiao et al., 2019). In humans, 17 Hsp70 genes and 30 pseudogenes have been discovered, some of which have up to 90 % sequence similarity (Brocchieri et al., 2008; Radons, 2016).

Hsp70 proteins are found in the cytosol of Archaea and Eubacteria, and in various compartments of eukaryotic cells (i. e. the nucleus, cytoplasm, mitochondria, chloroplasts, and endoplasmic reticulum) (Sarkar et al., 2011; Rosenzweig et al., 2019). The functions of Hsp70 include protein folding, disaggregation of protein aggregates, maintaining aggregation- prone proteins in an unfolded state, and participation in the translocation of proteins across the organelle membranes (Saibil, 2013; Bar-Lavan et al., 2016).

Hsp70 chaperones consist of a conserved N-terminal nucleotide-binding domain of ~44 kDa and a less conserved substrate-binding domain of ~30 kDa, connected by a short conserved hydrophobic linker (Sarkar et al., 2011; Bar-Lavan et al., 2016; Larburu et al., 2020). A disordered C-terminal tail of Hsp70 chaperones has variable length; in eukaryotic nuclear and cytosolic Hsp70 proteins, this region often contains the negatively charged motif Glu-Glu-Val-Asp, which interacts with specific cofactors, including Hsp40 co-chaperones (Rosenzweig et al., 2019). The nucleotide-binding domain consists of four subdomains that are arranged into two lobes separated by a deep crevice, in which an ATP-binding catalytic centre is located (Rosenzweig et al., 2019). The substratebinding domain contains two subdomains, α and β (Larburu et al., 2020). Subdomain β consists of β-sandwich folds and contains a hydrophobic substrate-binding pocket; subdomain α consists of α-helices and acts as a “lid” that closes over the substrate-binding pocket (Larburu et al., 2020).

**Fig. 1.**
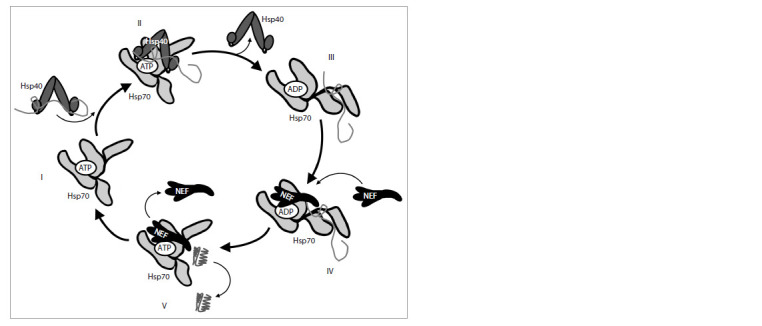
The chaperoning cycle of Hsp70. I – in the presence of ATP in the nucleotide-binding domain of Hsp70, the substrate-binding pocket of the chaperone is open and can bind misfolded proteins. II – co-chaperone Hsp40 delivers a client protein and stimulates ATP hydrolysis in the nucleotide-binding domain of Hsp70. III – ATP hydrolysis leads to the conformation change of the chaperone, resulting in the detachment of the substrate-binding domain subunits from the nucleotide-binding domain and their closure around the client protein. IV – co-chaperone NEF exchanges ADP for ATP in Hsp70’s nucleotide-binding domain. V – the substratebinding pocket of Hsp70 opens, releasing the correctly folded client protein.

The client protein binding and release is mediated by ATP binding and hydrolysis; the speed of these processes is regulated by co-chaperones Hsp40 (DnaJ in prokaryotes) and NEF (nucleotide exchange factor; GrpE in bacteria) (Saibil, 2013; Larburu et al., 2020). The reaction cycle of Hsp70 and its co-chaperones is shown in Figure 1. Binding of ATP to the catalytic centre of Hsp70’s nucleotide-binding domain causes the rotation of the domain’s subunits resulting in the inter-domain linker and the subunits of the substrate-binding domain attaching to the nucleotide-binding domain, opening the substrate-binding pocket (Rosenzweig et al., 2019). The substrate-binding domain of Hsp70 interacts with a short motif of the client protein, consisting of five hydrophobic amino acid residues flanked by charged residues (Larburu et al., 2020). This motif is present in virtually all proteins, which provides a great flexibility in substrate selection (Larburu et al., 2020).

Binding of the client protein induces ATP hydrolysis, which leads to the detachment of the linker and the substrate-binding domain subunits from the nucleotide-binding domain, and to the closure of the substrate-binding pocket with the client protein trapped in it (Rosenzweig et al., 2019; Larburu et al., 2020). Typically, ATP hydrolysis in the Hsp70 catalytic centre occurs at the rate of one molecule per 20–30 min; however, the involvement of Hsp40 co-chaperone that delivers the substrate to Hsp70 accelerates the reaction more than 1,000-fold (Bar- Lavan et al., 2016; Larburu et al., 2020). The ADP-to-ATP exchange is stimulated by NEF (Bar-Lavan et al., 2016). This process leads to the opening of the substrate-binding pocket of the chaperone and the release of the client protein (Fig. 1) (Larburu et al., 2020).

There are different hypotheses on the mechanism of protein folding by Hsp70 chaperones. Some models suggest that the binding of Hsp70 to hydrophobic regions of misfolded proteins protects them from aggregation, and upon release, the substrate undergoes folding independently (Bar-Lavan et al., 2016). Other models suggest that clamping of misfolded proteins in the substrate-binding pocket of one or several Hsp70 molecules promotes their unfolding (Bar-Lavan et al., 2016). Given that the Hsp70 chaperones not only prevent aggregation and promote folding, but also reactivate aggregated proteins, models defining unfolding as the main function of Hsp70 may be preferable (Bar-Lavan et al., 2016).

## Hsp60

The Hsp60 proteins (GroEL in bacteria, HSPD in humans), also known as chaperonins (Hemmingsen, 1992), are essential for the majority of organisms not only during stress, but also under normal conditions (Sarkar et al., 2011; Fan et al., 2020). Unlike Hsp70 and Hsp100 chaperones that mainly renature misfolded proteins and resolubilise protein aggregates, Hsp60 chaperonins are crucial for de novo protein folding (Saibil, 2013). Approximately 30 % of all E. coli de novo synthesised proteins adopt correct conformation through GroEL (Koumoto et al., 2001).

The DNA sequence of Hsp60 chaperonins is highly conserved, which makes it useful for phylogenetic analysis and identification of living organisms (Sarkar et al., 2011). Based on the structure and DNA sequence similarity, the chaperonins are divided into two groups (Saibil, 2013; Bar- Lavan et al., 2016). Group I includes the bacterial GroEL and its co-chaperonin GroES, the mitochondrial Hsp60 and its co-chaperonin Hsp10, and Cpn60 found in chloroplasts, alongside its co-chaperonin Cpn20 (Sarkar et al., 2011; Saibil, 2013; Zhang et al., 2016). Group II includes the archaeal thermosome and CCT (chaperonin-containing TCP1, also known as TriC) found in the cytoplasm of eukaryotes (Saibil, 2013). Hsp60 chaperonins form symmetrical structures comprising two back-to-back rings of 7 (Group I) or 8–9 (Group II) 60 kDa subunits each (Lopez et al., 2015). The Hsp60 subunits of both groups have three domains, namely, the apical,equatorial, and intermediate. The equatorial domain contains ATP-binding sites and establishes the interactions between the Hsp60 rings; the apical domain binds the substrate and, in Group I chaperonins, also Hsp10 co-chaperonins (Sarkar et al., 2011; Saibil, 2013; Bar-Lavan et al., 2016). 

Hsp10 co-chaperonins are homoheptameric structures composed of subunits of ~10 kDa, which bind to the rings of Group I chaperonins, closing their cavities like a lid (Saibil, 2013). In Group II chaperonins, the function of Hsp10 cochaperonins is carried out by an extra region of the apical domain (Saibil, 2013). The intermediate domain of Hsp60 monomers connects the apical and equatorial domains and undergoes a conformational change upon ATP binding, which facilitates the switching between the “open” state, with the inner surface of the cavity formed by the Hsp60 ring being hydrophobic, and the “closed” state, with the inner surface being hydrophilic (Sarkar et al., 2011; Saibil, 2013; Bar-Lavan et al., 2016).

**Fig. 2.**
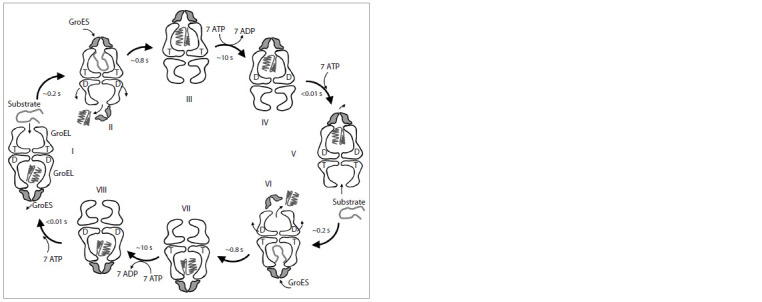
The reaction cycle of Hsp60 using the example of bacterial GroEL and its co-chaperonin GroES (homoheptamers of GroEL and GroES are shown in cross-section). I – hydrophobic regions of a misfolded client protein interact with the hydrophobic inner surface of the apical domains of the ATP-bound open ring of GroEL (top in the image). II – the GroEL cavity is rapidly (within ~0.2 s (Horwich, 2011)) closed by the GroES co-chaperonin, and the substrate becomes “trapped” inside the GroEL cavity. III – the binding of GroES causes the apical domains of GroEL to rotate 100° clockwise (Horwich, 2011; Clare et al., 2012), which results in the inner surface of the GroEL ring becoming hydrophilic, promoting the folding of the substrate. IV – approximately 10 s later, ATP hydrolysis occurs in the equatorial domains of the ring containing the substrate protein, leading to the weakening of the interaction between the two rings allowing the second ring to bind ATP and begin the folding cycle (Horwich, 2011). V – the GroES co-chaperonin dissociates from the first GroEL ring; the folding cycle of a new substrate protein begins in the second ring. VI – the folded (or still misfolded) client protein leaves the now open cavity of the first ring; ADP and phosphate dissociate from the equatorial domains of the first GroEL ring. VII, VIII – the first ring is unable to bind ATP and, therefore, the new substrate protein, until ATP hydrolysis occurs in the second ring. T – ATP; D – ADP.

The reaction cycle of Group I chaperonins GroEL/GroES is described in Figure 2. The folding cycle performed by Group II chaperonins follows the same pattern, except for the cavity closure being performed not by Hsp10 but by the extra regions of the apical domains as a result of ATP hydrolysis, and the rings of Group II chaperonins switching between the “open” and “closed” states synchronously, rather than sequentially (Kumar et al., 2015; Lopez et al., 2015).

The substrates of Hsp60 include peptides with a molecular mass between 35 and 60 kDa, with the maximum substrate size determined by the volume of the Hsp60 ring cavity, which in GroEL is ∼175,000 Å3 (Bar-Lavan et al., 2016). The chaperonins perform the folding of such essential proteins as actin and tubulin in eukaryotes, and the RbcL subunit of the RuBisCO enzyme, which participates in the Calvin cycle (Sarkar et al., 2011; Hayer-Hartl, 2017).

## Small HSPs

Small HSPs (HSPB in humans) have molecular masses between 12 and 43 kDa (Sarkar et al., 2011). In eukaryotes, they are localised to the cytoplasm, nucleus, mitochondria, chloroplasts, and peroxisomes. Prokaryotes and unicellular eukaryotes typically have one or two cytosolic small HSPs, although some bacteria may have several (for example, bacteria of the genus Bradyrhizobium can have up to eight small HSP genes) (Mogk et al., 2019). In multicellular eukaryotes, the number of genes encoding small HSPs ranges from 10 in humans to 50 in higher plants (Mogk et al., 2019).

Unlike other HSP families, small HSPs do not have the ability to refold denatured and aggregated proteins, and they do not hydrolyse ATP when performing their functions, the main of which is to prevent the aggregation of misfolded proteins (Bar-Lavan et al., 2016; Mogk et al., 2019). In addition to the prevention of protein aggregation, small HSPs are involved in several key physiological processes, such as cellular differentiation and apoptosis (Fu, 2015).

A characteristic feature of small HSP family members is the presence of a conserved α-crystallin domain, named after the eye lens protein of vertebrates, α-crystallin (Sarkar et al., 2011; Bar-Lavan et al., 2016; Paul et al., 2016; Mogk et al., 2019). This domain consists of 90–100 amino acid residues forming a β-sandwich consisting of 7–8 antiparallel β-strands (Mogk et al., 2019). The α-crystallin domain of small HSPs is surrounded by less conserved N- and C-terminal domains (Mogk et al., 2019). The N-terminal domains are especially diverse in amino acid composition and size, consisting of 24–247 amino acid residues (56 on average) (Mogk et al., 2019). The C-terminal domains are up to 20 amino acid residues long (on average, 10) and in 90 % of small HSPs contain the conserved motif Ile-X-Ile/Val (IXI/V) that plays a key role in the oligomerisation of small HSPs (Saji et al., 2008; Mogk et al., 2019).

The oligomers of small HSPs are hollow spheres consisting of 12–32 protomers (Saji et al., 2008; Mogk et al., 2019). Each protomer is a dimer of small HSPs, and the oligomers can be composed of a single type or several types of small HSPs (Mogk et al., 2019). The dimerisation of small HSPs occurs through the interaction of their α-crystallin domains, and the oligomerisation is established by the N- and C-terminal domains (Mogk et al., 2019). Small HSP oligomers are dynamic and constantly exchange dimers (Żwirowski et al., 2017). During stress (e. g. temperature fluctuations, ion balance disruption, pH changes), the equilibrium shifts towards the formation of smaller oligomers and dissociation into dimers, which bind to the hydrophobic regions of misfolded proteins (Żwirowski et al., 2017; Mogk et al., 2019).

**Fig. 3.**
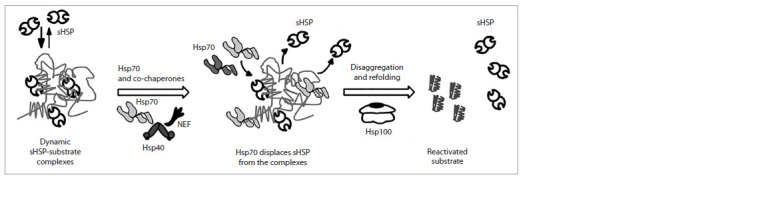
Schematic model of small heat shock protein release from complexes with substrate proteins by Hsp70 chaperones and the following reactivation of the substrate proteins by Hsp70 and Hsp100 chaperones. The complexes of small HSPs with substrates consist of a stable core formed by misfolded proteins and the associated small HSPs, and of a dynamic shell, on the surface of which small HSPs bind to and dissociate from the complex (the equilibrium is shifted toward binding). Hsp70 chaperones displace small HSPs from the complexes, releasing them, and then present the substrate proteins to Hsp100 chaperones, which then pull them through their central pore and reactivate the substrates.

Small HSPs have low specificity and interact with a wide range of different substrate proteins (Mogk et al., 2019). When binding to misfolded proteins, small HSPs form complexes consisting of two layers – a stable core containing small HSPs and immobile substrate proteins, and a dynamic shell of small HSPs, which alternately bind to and dissociate from the complex (Fig. 3) (Żwirowski et al., 2017). The size of these complexes is smaller than that of the misfolded protein aggregates, but their molecular mass usually exceeds 1 MDa (Żwirowski et al., 2017).

The size of small HSP-substrate complexes depends on the small HSP-to-substrate ratio; the higher the proportion of small HSPs, the smaller the complex size. At sufficiently high levels of small HSPs, the complexes become soluble (Mogk et al., 2019). At low concentrations of small HSPs, the substrates form dense, insoluble complexes, but unlike insoluble aggregates formed solely by misfolded proteins, the substrates are maintained in a conformation that allows chaperones from other HSP families to extract them from the complex and reactivate them (Mogk et al., 2019).

The reactivation of such denatured proteins is carried out by Hsp70 chaperones (Fig. 3) (Żwirowski et al., 2017). Through competitive substrate binding, Hsp70 chaperones displace small HSPs from the complex shell and perform substrate folding, sometimes in cooperation with Hsp100 (Żwirowski et al., 2017).

The shell of the complexes has several important physiological functions: (1) it maintains the size and solubility of the complex by protecting the hydrophobic regions of the substrate proteins from interacting with other misfolded proteins, thereby preventing their aggregation; (2) it forms a selective barrier that is permeable only to Hsp70 chaperones; (3) it increases the demand for Hsp70 chaperones, in comparison with protein aggregates (Żwirowski et al., 2017).

Thus, although small HSPs keep aggregation-prone proteins in a state suitable for folding, they impede substrate reactivation at insufficient Hsp70 concentrations. This mechanism may allow for the isolation of misfolded proteins under stress conditions, when the number of available Hsp70 chaperones is limited, for example, during chronic stress (Żwirowski et al., 2017). At the same time, at low Hsp70 concentrations, small HSPs may promote the formation of protein aggregates because they prevent the ubiquitination of substrate proteins in the complex and their degradation (Mogk et al., 2019).

## Conclusion

As molecular chaperones, heat shock proteins play a central role in the maintenance of protein homeostasis and are essential for all known prokaryotes and eukaryotes. Members of different HSP families perform various functions, assisting in the correct folding of polypeptides, preventing the aggregation of misfolded proteins, carrying out their refolding, or promoting their degradation. To perform all these functions, molecular chaperones work in tandem with each other and with numerous co-chaperones, maintaining the cell’s proteome in working condition. Thus, heat shock proteins are the “first line of defence” for cells against the toxic effects of damaged and misfolded proteins, both under normal conditions and when exposed to various stressors.

## Conflict of interest

The authors declare no conflict of interest.
